# Advancing precision medicine in gliomas through single-cell sequencing: unveiling the complex tumor microenvironment

**DOI:** 10.3389/fcell.2024.1396836

**Published:** 2024-08-02

**Authors:** Jinwei Li, Yang Zhang, Cong Liang, Xianlei Yan, Xuhui Hui, Quan Liu

**Affiliations:** ^1^ Department of Neurosurgery, West China Hospital, Sichuan University, Chengdu, Sichuan, China; ^2^ Department of Neurosurgery, Liuzhou Workers Hospital, Liuzhou, Guangxi, China; ^3^ Graduate School of Medicine, Kunming Medical University, Kunming, Yunnan, China; ^4^ Department of Pharmacy, Liuzhou Workers Hospital, Liuzhou, Guangxi, China

**Keywords:** glioblastoma, TME, single-cell, GAMs, TAMs

## Abstract

Glioblastoma (GBM) displays an infiltrative growth characteristic that recruits neighboring normal cells to facilitate tumor growth, maintenance, and invasion into the brain. While the blood-brain barrier serves as a critical natural defense mechanism for the central nervous system, GBM disrupts this barrier, resulting in the infiltration of macrophages from the peripheral bone marrow and the activation of resident microglia. Recent advancements in single-cell transcriptomics and spatial transcriptomics have refined the categorization of cells within the tumor microenvironment for precise identification. The intricate interactions and influences on cell growth within the tumor microenvironment under multi-omics conditions are succinctly outlined. The factors and mechanisms involving microglia, macrophages, endothelial cells, and T cells that impact the growth of GBM are individually examined. The collaborative mechanisms of tumor cell-immune cell interactions within the tumor microenvironment synergistically promote the growth, infiltration, and metastasis of gliomas, while also influencing the immune status and therapeutic response of the tumor microenvironment. As immunotherapy continues to progress, targeting the cells within the inter-tumor microenvironment emerges as a promising novel therapeutic approach for GBM. By comprehensively understanding and intervening in the intricate cellular interactions within the tumor microenvironment, novel therapeutic modalities may be developed to enhance treatment outcomes for patients with GBM.

## 1 Introduction

Gliomas represent the most prevalent primary central nervous system cancers in adults. The current standard treatment approach involves a combination of maximal safe surgical resection along with adjuvant radiotherapy and chemotherapy (Zhao et al., 2021). The median survival is less than 15 months as the tumors always recur in a very short time (Stupp et al., 2005; Zhao et al., 2021). In recent years, advancements in technologies such as whole exome sequencing, Cytometry by time-of-flight (CyTOF), and single-cell and spatial transcriptomics have shed light on the intricate interactions between tumor cells and the tumor microenvironment (TME). These studies have revealed that the complex interplay between tumor cells and the TME significantly contributes to the heterogeneity and poor prognosis of tumors (Klemm et al., 2020; Batchu et al., 2023; Nettnin et al., 2023). Glioblastomas (GBM), known for their characteristic diffuse infiltrative growth, have been found to recruit normal cells from their surrounding environment to support tumor growth, maintenance, and invasion into the brain (Broekman et al., 2018). Furthermore, a crucial factor in driving the malignant growth of glioblastoma is the communication and manipulation of information between tumor cells and other cells in the brain microenvironment. This intricate interaction plays a significant role in promoting the growth of tumor cells and their acquisition of resistance to treatment (Broekman et al., 2018). Given the unsatisfactory outcomes of current treatments for gliomas, numerous researchers have focused on comprehensively investigating the development of GBM within its TME. These research endeavors are anticipated to uncover novel targets and therapeutic strategies for the effective treatment of tumors.

Immune checkpoint blockade (ICB), adoptive cell therapy, and vaccines are among the primary immunotherapies used for tumors. Immunotherapy has emerged as a beacon of hope in cancer treatment, revolutionizing the prognosis for numerous patients with advanced solid tumors like melanoma, non-small cell lung cancer, and renal cell carcinoma (Braun et al., 2021; Guo et al., 2021; Ji et al., 2022). Bevacizumab is one of the targeted drugs approved for the treatment of recurrent glioblastoma in China. However, current studies have shown that bevacizumab fails to achieve the desired efficacy in newly diagnosed glioblastoma patients (Wefel et al., 2021). A phase III study comparing a PD-1 inhibitor (Nivolumab) with bevacizumab in the treatment of recurrent glioblastoma showed that Nivolumab did not improve overall survival, progression-free survival, or overall response rate in patients with recurrent glioblastoma (Reardon et al., 2020). Immunosuppressive signals from gliomas *in vitro* and *in vivo* can directly regulate the function of immune cells, including Mesenchymal stem cells and tumor-related macrophages, to promote immunosuppression (Mi et al., 2020; Qi et al., 2022). Immune tolerance plays a crucial role in dampening the initiation of an immune response in GBM. Despite ongoing efforts, current immunotherapies have demonstrated limited effectiveness in treating adult GBM. Reversal of T-cell depletion by cell-specific modulation, combined with alterations in immunosuppressive TME, may have a profound impact on the efficacy of immunotherapy in patients with malignant glioma (Watowich et al., 2023). Therefore, it is imperative to delve deeper into the cellular interactions within the tumor microenvironment associated with GBM to uncover novel therapeutic avenues.

Recent advancements in multi-group sequencing have facilitated numerous discoveries and studies on the intricate interactions among cells within the tumor microenvironment of GBM. Consequently, this review aims to comprehensively analyze the impact of diverse cell subsets in brain GBM on tumor growth and treatment by synthesizing findings from previous literature.

### 1.1 Unveiling the complexity of glioma tumor microenvironment through single-cell sequencing

The utilization of single-cell sequencing (scRNA-seq) has represented a significant advancement in the study of gliomas. Glioma, a prevalent and highly heterogeneous brain tumor, often poses challenges to conventional cancer research methodologies. Traditional approaches typically involve analyzing the average characteristics of a large population of tumor cells, overlooking both intercellular and intracellular variabilities. In contrast, scRNA-seq technology enables a comprehensive exploration of cellular heterogeneity by scrutinizing the gene expression of individual cells. Furthermore, by examining the gene expression regulatory network among different cell types, scRNA-seq can unveil the mechanisms underlying interactions between tumor cells and immune cells, endothelial cells, and other cellular components within the tumor microenvironment. The single-cell assay for transposase-accessible chromatin (scATAC-seq) can help us to understand the transcriptional and regulatory processes of cells at the genomic level, reveal the sites of different regulatory factors, and analyze the gene information from the perspective of epigenetics. scATAC-seq has great potential for applications in open chromatin mapping, cell differentiation, and development, disease pathogenesis, tumor microenvironment, biomarkers, etc. (Buenrostro et al., 2015). Single-cell sequencing requires the dissociation of cells, which results in the loss of information about the original location of the cells in the tissue, so it is difficult to map detailed cellular maps and relationships between cells at the level of tissue architecture. This is where the spatial transcriptome can be used to help understand the cellular and organizational functions of multicellular organisms, mapping cells from their physiological, morphological, and anatomical contexts, as well as their spatial architecture (Jung et al., 2022). This enhanced understanding of the cellular composition and interactions in gliomas holds promise for advancing precision medicine approaches and developing targeted therapies for this complex and challenging cancer.

**FIGURE 1:**
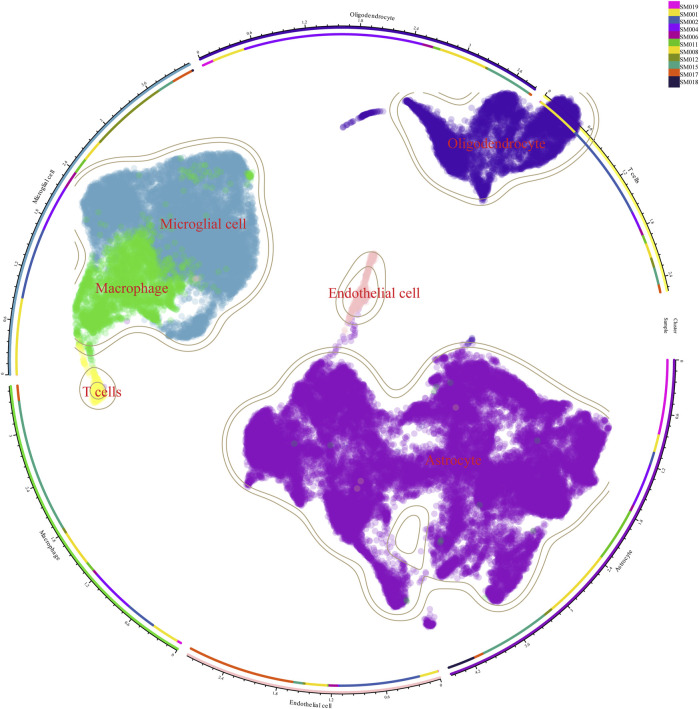
Cellular clustering after single-cell sequencing of tumor tissue from 11 glioma patients. There were 11 glioma samples divided into macrophages, microglia, oligodendrocytes, astrocytes, and T cells.

**FIGURE 2:**
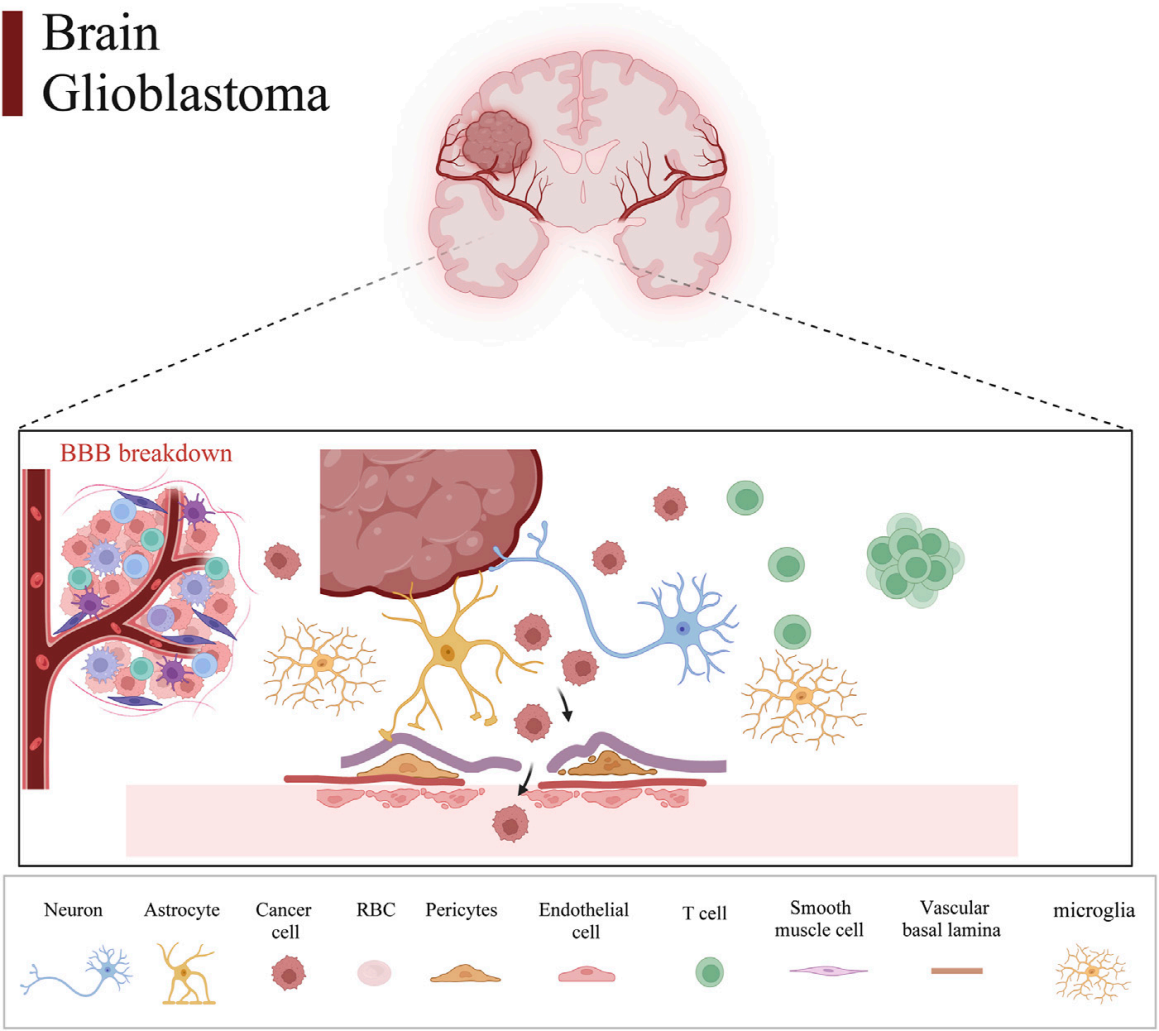
Glioma tumor microenvironment and the blood-brain barrier. Intracranial glioma cells comprise a variety of cells and tissues that make up the blood-brain barrier, which consists of neurons, astrocytes, tumor cells, erythrocytes, pericytes, endothelial cells, T cells, smooth muscle cells, vascular basal lamina, and microglia.

Therefore, we integrated several publicly available scRNA-seq GBM datasets for data processing and normalization. Data from Synapse database (https://synapse.org/singlecellglioma) (Johnson et al., 2021). We used a total of 11 glioma patients with a total cell count of 55,089. The total number of Astrocytes, Microglial cells, macrophages, T cells, Endothelial cells, and Oligodendrocytes after our clustering and annotation (Figure 1). We found that Microglial cells, Macrophages, and Oligodendrocytes occupied the majority (Figure 1). The number of immune T-cells was very low, probably due to confinement to the bone marrow (Chongsathidkiet et al., 2018). Gliomas are commonly referred to as “cold tumors” due to their immunosuppressive microenvironment, which impedes the infiltration and function of immune cells that could potentially target and eradicate tumor cells. The glioblastoma microenvironment is a complex milieu comprising various elements that interact dynamically to influence tumor development and progression. This microenvironment encompasses tumor cells, the extracellular matrix (ECM), blood vessels, and a diverse array of immune cells, including monocytes, macrophages, mast cells, microglia, neutrophils, and T cells (Broekman et al., 2018). As in Figure 2, the Blood-brain barrier and tumor microenvironment are closely related. Immune cells play an important role in tumor immune escape and anti-tumor immune response. Macrophages from the hematopoietic system are located in the tumor tissue due to the attraction of chemical factors secreted by tumor cells. They play an important role in inflammation and TME, which can not only promote tumor growth and spread but also exert an anti-tumor effect (Mei et al., 2023).

In essence, the tumor microenvironment (TME) of gliomas comprises an intricate network of various cellular and non-cellular elements that interact and collectively govern tumor growth and advancement. A comprehensive comprehension of the evolving dynamics within the tumor microenvironment and the diverse immune milieu within a genetic framework is crucial for the advancement of novel therapeutic approaches for tumors.

### 1.2 Interactions between tumor cells and cells of the tumor microenvironment

Hierarchical clustering analysis of inferCNV and CopyKAT results have identified the majority of vascular endothelial and mural cells as diploid non-tumor cells. This suggests that in glioblastoma (GBM), tumor cells derived from neural stem/precursor cells tend to maintain their neural lineage restriction and are less likely to differentiate into non-neural lineage cells. The analysis indicates that different malignant cells within GBM converge into four distinct cell states: neural progenitor cell-like (NPC-like), oligodendrocyte progenitor cell-like (OPC-like), astrocyte-like (AC-like), and mesenchymal stromal cell-like (MES-like) states. These cell states exhibit potential for plasticity, allowing for transitions between different states within the tumor microenvironment (Hara et al., 2021). These types of tumor cells play a crucial role in driving clonal evolution, tumor progression, and treatment resistance (Greenwald et al., 2024). Studies have shown that AC-like tumor cells are associated with poor prognosis in patients (Zheng et al., 2023). He dispersion and connection of AC-like cells with other transcriptional subtypes are associated with a reduced risk (Greenwald et al., 2024). At the same time, NPC-like cells couple with neurons in the infiltrating regions, mimicking the migration of normal NPCs to developing neurons (Greenwald et al., 2024). Pseudotime analysis revealed the dynamic transition of tumor cells from pro-neural to mesenchymal subtypes (Xiong et al., 2022). Urthermore, patients with higher MES-like scores always correlate with worse prognosis compared to those with lower MES-like scores (Xiong et al., 2022).

Glioblastoma stem cells (GSCs) and the immunosuppressive tumor microenvironment are two important factors contributing to glioblastoma genesis, treatment resistance, and recurrence (Pang et al., 2022). It has been found and characterized that GSCs-derived TFPI2 can regulate the maintenance of tumor stem cell stemness and the migration and immunosuppression of microglia in the tumor microenvironment through different mechanisms, respectively (Pang et al., 2023). The tumor mesenchymal-like state is associated with increased abundance and cytotoxicity of tumor-infiltrating T cells (Hara et al., 2021). A large number of MES subtypes correlate with macrophage abundance (Wang et al., 2017). In glioblastoma, the MES status of macrophages and cancer cells may also represent a therapeutic opportunity, as they are associated with high levels of MHC-I and MHC-II as well as a high number of T-cells, favoring a cytotoxic state, which may affect the response to immunotherapy (Hara et al., 2021). Infiltration of glioma-associated macrophages and microglia (GAMs) is usually triggered by chemokines secreted by GBM cells. Simultaneous environment-dependent tumor-immune symbiotic interactions promote tumor progression and treatment resistance in GBMs (Pang et al., 2022). Immunosuppressive GAM releases different cytokines and growth factors, such as IL-6, IL-11, IL-1β, IL-10, and TGF-β1, to promote GBM progression by activating pre-tumor signaling in GBM cells (Ye et al., 2012; Bloch et al., 2013; Zhang et al., 2018; Li et al., 2021; Liu et al., 2021; Ochocka et al., 2021).

In conclusion, the interactions between tumor cells and immune cells within the tumor microenvironment play a crucial role in driving the growth, infiltration, and metastasis of gliomas. These interactions also impact the immune status of the tumor microenvironment and influence the response to therapeutic interventions. By gaining a deeper understanding of these complex interaction mechanisms, researchers can identify novel therapeutic targets and develop more effective treatment strategies for gliomas. This knowledge can ultimately lead to improved outcomes for patients with glioma and other types of cancer.

### 1.3 Microglia and macrophages in the microenvironment with glioblastoma

#### 1.3.1 Origin and function of microglia and macrophages

The blood-brain barrier is crucial as a natural barrier to the central system. In tumors, up to 30% of these cells, most of the activated GAMs serve to promote tumor formation and produce immunosuppression (Hambardzumyan et al., 2016). The Tumor-associated macrophages (TAMs) are mainly derived from peripheral blood monocytes and tissue-resident macrophages (Christofides et al., 2022). In adulthood, the other major source of macrophages in tissues is peripheral blood monocytes, in addition to long-surviving and self-renewing tissue-resident macrophages. Tissue-resident macrophages are also present in the central nervous system, including microglia in the parenchyma and macrophages in the perivascular spaces at the borders of the brain parenchyma, the dura mater, and the choroid plexus. Microglia are directly derived from red myeloid precursor cells of the yolk sac during embryonic development (Gomez Perdiguero et al., 2015). In addition to its functions of regulating neuronal survival and apoptosis, assisting in axonal growth, and neuronal migration, it also acts primarily as a resident immune cell in the brain. Maintain the integrity of the blood-brain barrier by constantly lengthening and contracting their cell protuberances to scan the central nervous system, mediate immune responses, and cooperate with astrocytes (Qiang et al., 2020). Another key reason why current targeted antineoplastic therapy cannot provide a lasting response in GBM is the adaptability of TME. Most of the non-tumor cells in GBM’s TME are innate immune cells called macrophages in both human and mouse GBM models. Microglia exercising immune functions in the CNS play an important role in the itinerary of the glioma immune microenvironment.

#### 1.3.2 The role of GAMs in GBM

**FIGURE 3:**
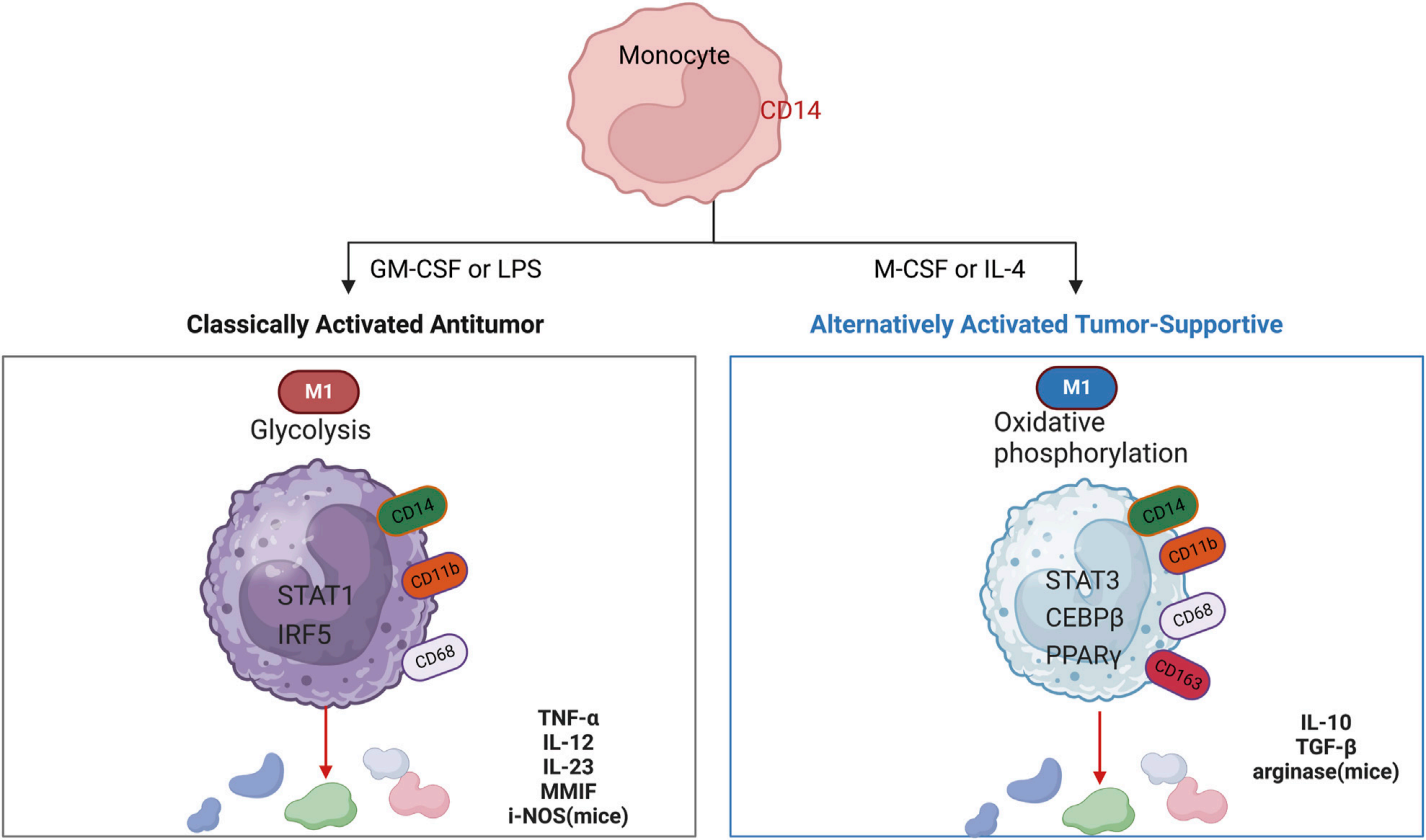
Cytokines that induce polarized M1 and M2 macrophages, markers for their identification, key transcriptional pathways (nucleus), metabolism (cytoplasm), and secreted immune effectors. Schematic showing cytokines used to induce polarized M1 and M2 macrophages.

Macrophages are the main non-tumor infiltrating substances in the microenvironment of glioblastoma. Macrophages induce the transformation of glioblastoma cells into mesenchymal-like cells, which is related to the increase of mesenchymal procedures of macrophages and the enhancement of cytotoxicity of T cells (Hara et al., 2021). Macrophage aggregation correlates with malignancy in human gliomas, which supports a therapeutic target for GAMs (Hussain et al., 2006; Komohara et al., 2008). In the early stages of gliomas, tumor-associated macrophages (TAMs) exhibit a pro-inflammatory M1 phenotype, which inhibits tumor proliferation. However, in advanced gliomas, TAMs predominantly display an M2 phenotype, known for inducing an immunosuppressive response and enabling immune escape from the tumor (Qi et al., 2020). GBM-induced M2-like macrophages showed increased secretion of anti-inflammatory cytokines TGF- β one and IL-10, which promoted capillaries, proliferation, and angiogenesis sprouting of vascular endothelial cells, while classic activated M1-like macrophages inhibited angiogenic activity (Cui et al., 2018). Radiation therapy itself causes changes in the tumor microenvironment that may make the tumor more aggressive. Radiation therapy induces a rapid inflammatory response that leads to the recruitment of GAMs. Moreover, the M1 phenotype is more sensitive to radiation than the M2 phenotype, so M2 macrophages are more resistant than M1 macrophages after radiation therapy (Leblond et al., 2017). In the presence of activated glioma-derived factors (i.e., conditions mimicking the late stage of pathology), microglia showed a mixture of polarized phenotypes (M1 and M2a/b), with inducible nitric oxide synthase, arginase, and IL-10^42^. M1 and M2 types of macrophages are stimulated by different substances and produce different effects, respectively (Wei et al., 2020) (Figure 3). Moreover, a large number of CD68-positive cells were detected in the surgically resected human glioma tissues of GBM patients, which was a sign of phagocytic activity of macrophages/microglia (Lisi et al., 2014). The number of macrophages expressing CD68 increases with the grade of glioma, which is often associated with a negative impact on survival prognosis (Komohara et al., 2008). Macrophage typing is usually divided into M1 and M2 types, but macrophage typing is different in single-cell analysis. A clear division between M1 and M2 macrophage subtypes does not exist. Macrophage typing in single cells is usually based on the signaling pathways they act on or on highly expressed markers.

GAMs are the most abundant immune cells in the glioma microenvironment (Yang et al., 2023). He cooperation between CD4^+^ T cells and microglia is essential for generating an effective response to immune checkpoint blockade therapy in GBM (Chen et al., 2023). Studies have demonstrated that targeting TAMs can inhibit PDGFB-driven glioma growth (Rao et al., 2022). Research has shown that single-cell sequencing of glioblastoma multiforme (GBM) reveals that microglia are in a state of severe oxidative stress, inducing NR4A2-dependent transcriptional activity in these cells. The transcriptional mechanisms regulated by NR4A2 alter cholesterol metabolism, leading to an alternatively activated phenotype in CD8^+^ T cells and impaired antigen presentation capability within GBM (Ye et al., 2023). Additionally, oxidative stress induces an alternatively activated state in microglia and disrupts their antigen-presenting function to CD8^+^ T cells. Longitudinal analysis of GBM progression in mouse models indicates that early-stage GBM is primarily composed of microglia, whereas late-stage GBM shows increased infiltration of macrophages (Yeo et al., 2022).

In summary, glioma-associated microglia/macrophages (GAMs) are cell populations closely linked to GBM and play a significant role in the development of GBM by facilitating tumor growth and invasion into the normal brain tissue. Recent research has highlighted GAMs as a promising target for targeted therapy in GBM treatment. However, the challenge remains in translating these experimental findings into clinical applications. As our understanding of the role of GAMs in GBM continues to evolve, it is expected to offer new insights and potential avenues for clinical treatment strategies in the future. Efforts to harness the therapeutic potential of targeting GAMs hold promise for improving outcomes in GBM patients.

### 1.4 T-cell immunity and the glioblastoma microenvironment

**FIGURE 4:**
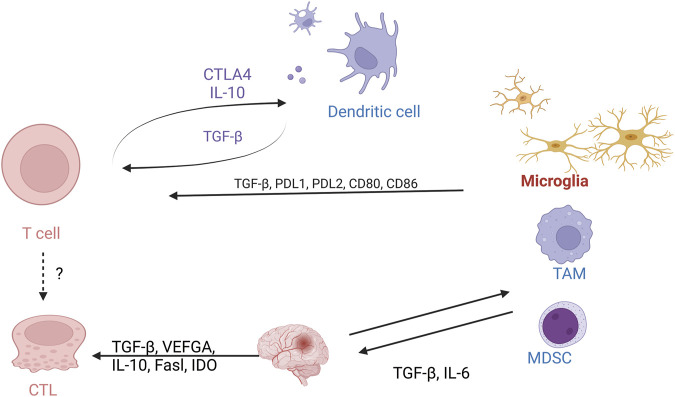
Cellular interactions of T cells with the glioma tumor microenvironment. Schematic demonstrating the interaction between T cells on macrophages, microglia, and MDSC cells.

**FIGURE 5:**
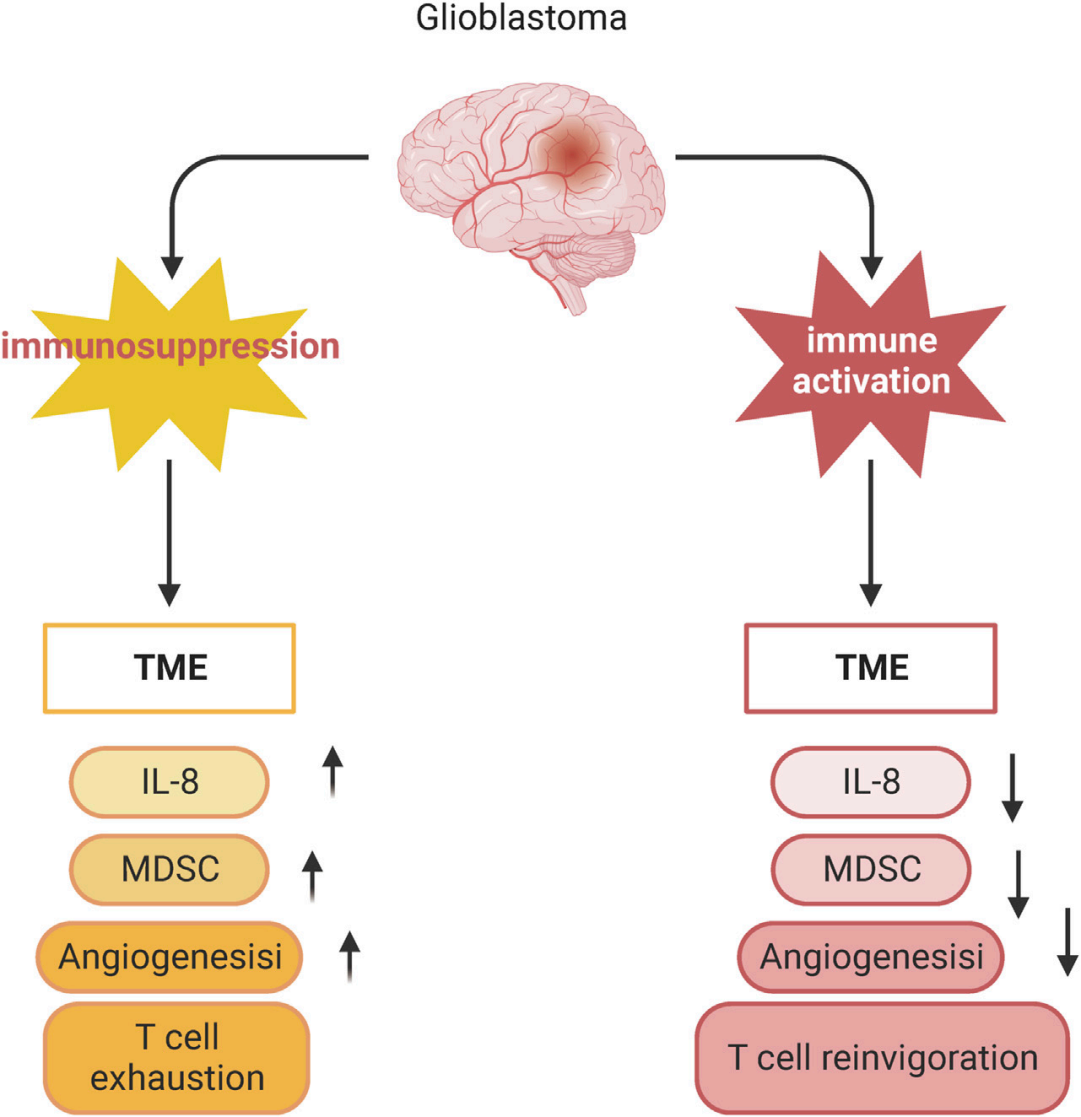
Blocking IL-8 reverses the immunosuppressive microenvironment of tumors. Schematic showing changes in T cell phenotype in suppressed and activated tumor microenvironments.

T-cell immunity plays an important role in the glioma TME (Figure 4). However, the anti-tumor function of T cells is often limited due to the immune escape mechanisms and immunosuppressive environment of gliomas (Ravi et al., 2022). The limitations of GBM immunotherapy are intricately linked to the deficiency of T-cells within the brain tumor microenvironment. A comprehensive analysis combining single-cell transcriptome and spatial transcriptome sequencing has shed light on the role of interleukin-10 (IL-10) secreted by myeloid cells in driving T-cell dysfunction within the GBM microenvironment. This IL-10-mediated T-cell dysfunction ultimately promotes tumor cell growth and suppresses the immune response against the tumor. Understanding the mechanisms by which IL-10 and myeloid cells contribute to T-cell deficiency in GBM can provide valuable insights for developing targeted immunotherapies that aim to overcome these barriers and enhance the efficacy of immune-based treatments for GBM (Ravi et al., 2022). It was demonstrated that after α-PD-1 treatment, regulatory T cells (Tregs) with upregulated CD103 + lipid metabolism accumulated in the tumor microenvironment and suppressed the immune checkpoint blockade response by inhibiting CD8 + T cell activation (van Hooren et al., 2023). Treg cell targeting triggers the formation of tertiary lymphoid structures, enhances CD4^+^ and CD8^+^ T cell frequency and function, and unleashes radio immunotherapeutic efficacy (van Hooren et al., 2023). The spatial distribution of tumor-associated myeloid cells (TAM) coincided with hypoxic regions in GBM while targeting hypoxic sites disrupted the spatial distribution of TAM (Sattiraju et al., 2023). One study identified a population of CD4^+^ T cells that highly express IL-8 (Liu et al., 2023). Blocking IL-8 was able to reverse the immunosuppressive microenvironment of the tumor and enhance the therapeutic effect of immune checkpoint blockade (Liu et al., 2023) (Figure 5).

### 1.5 Endothelial cell and glioblastoma microenvironment interactions

#### 1.5.1 Endothelial cells and the treatment of GBM

Persistent angiogenesis, characterized by the continuous formation of new blood vessels, is a key feature of cancer, including GBM. GBM is highly vascularized, with abnormal blood vessel structure and function, making it an attractive target for therapies that exploit this property. Antivascular therapies that focus on targeting vascular endothelial growth factor (VEGF) and its receptors have been developed and used in GBM treatment. VEGF affects endothelial cell proliferation, migration, and mitosis of vascular endothelial cells in gliomas, thereby promoting angiogenesis. Blood vessels are abnormally abundant in gliomas. Tumor cells and vascular endothelial cells in glioblastoma are often accompanied by high expression of VEGF-A and its receptor VEGFR2, which regulate vascular proliferation in an autocrine and paracrine manner (Michaelsen et al., 2018). However, the therapeutic effects of these approaches in GBM patients have been limited and short-lived. This could be attributed to the challenges of effectively eradicating tumor endothelial cells or inhibiting their functions in a sustained manner. Further research and innovative strategies are needed to overcome these limitations and improve the efficacy of antivascular therapies in treating GBM (Fan, 2019). Therefore, the effective eradication of tumor endothelial cells is very important for tumor therapy (Huang et al., 2020). Endothelial transformation is a cellular mechanism to control tumor chemoresistance. Targeting WNT-mediated endothelial transformation and stemness activation could be a next-generation antivascular therapy strategy for cancer (Huang et al., 2016; Fan, 2019). Targeting Wnt/β-catenin-mediated EC transformation and stemness activation may overcome therapeutic resistance in GBM (Huang et al., 2020). There is intercommunication between EC and tumor cells. Vascular leakage in glioblastoma is driven by two mechanisms: increased paracellular transport by altering tight junctions between endothelial cells or enhanced transcellular transport (Zeng et al., 2023). The specialization of endothelial cells is a critical feature of the blood-brain barrier (BBB). It is defined by the establishment of tight junctions between neighboring endothelial cells and the expression of transporters associated with the BBB. Recent single-cell sequencing studies have unveiled diverse expression patterns of junctional and transporter proteins across distinct endothelial cell clusters within the BBB (Xie et al., 2021).

#### 1.5.2 Transformation of endothelial cells with glioma stem cells

ECs are closely related to GSC (Calabrese et al., 2007; Lathia et al., 2011). Glioblastoma stem cells can differentiate into endothelial cells and form vascular structures, thus promoting tumor vascularization (Ricci-Vitiani et al., 2010). ECs produce a variety of growth factors that stimulate GSC self-renewal and tumorigenesis (Galan-Moya et al., 2011; Zhu et al., 2011; Infanger et al., 2013). GSCs can differentiate into ECs or pericytes to create their vascular endothelium (Wang et al., 2010; Guichet et al., 2015). GSC also produces various cytokines and chemokines, some of which are known to activate ECs (Thirant et al., 2012). At the same time, tumor-associated vascular cells and tumor-derived vascular endothelial fine were found to differ in molecular characteristics, with extensive heterogeneity between the two (Carlson et al., 2021). Moreover, tumor tissue produces rare endothelial cells (Carlson et al., 2021). The recurrence of GBM, as well as the powerful invasion, division, and angiogenesis of GBM itself, is strongly associated with GSCs. The process of radiotherapy, in turn, makes the tumor-derived endothelial cells more competent, contributing to the maintenance of GSCs, which can be said to mediate tumor recurrence (Deshors et al., 2019).

## 2 Challenges and prospects

While various sequencing methods and experimental approaches have been employed to investigate the immune microenvironment of glioblastoma (GBM), the significant heterogeneity among patients poses challenges for targeted treatments. Encouragingly, recent advancements in the study of pan-cancer immune cell profiles across different tumor types are shedding light on potential therapeutic strategies. For instance, T cells play a pivotal role as anti-tumor immune cells, with cytotoxic T cells being the primary effector cells responsible for eliminating cancer cells. Within the tumor microenvironment, tumor-infiltrating T cells include those that recognize and respond to tumor antigens. However, during tumorigenesis and progression, these T cells often undergo differentiation into a dysfunctional state, leading to T cell exhaustion (Chen and Flies, 2013). A related study established a single-cell RNA sequence pan-cancer map of T cells from 316 donors in 21 cancer types and identified different patterns of T cell composition (Zheng et al., 2021). It reveals the interrelationship between T-cell subpopulations and tumors. Moreover, this single-cell level pan-cancer T cell study has deepened the understanding of tumor-infiltrating T cells in many aspects, and will further promote the development of new cancer immunotherapies. Meanwhile, NK Cell Tumor Atlas performed a comprehensive single-cell RNA sequencing analysis of NK cells from 716 cancer patients (covering 24 cancer types) (Tang et al., 2023). Myeloid cell subsets, particularly LAMP3+ dendritic cells, appear to mediate the regulation of anti-tumor immunity in NK cells (Tang et al., 2023). Therefore, the next step would be to integrate the large number of single-cell sequencing results for GBM to construct a cellular map of the GBM immune microenvironment. This may help to explain the heterogeneity of the tumor and the potential clinical utility of finding new cell subpopulations as therapeutic targets.

The diffuse infiltrative nature of glioblastoma (GBM) renders complete surgical resection for a potential cure unattainable. Therefore, a multidisciplinary treatment approach combining various modalities is urgently required. Additionally, interactions within the tumor microenvironment can influence the efficacy of pharmacological interventions in GBM. For instance, the M1/M2 polarization of macrophages is a dynamic and reversible process that may impact drug resistance and immunosuppression in GBM. Further research is warranted to elucidate its role in GBM. Comparative multi-omics sequencing of GBM patients pre- and post-treatment represents a promising avenue for exploration.

## 3 Conclusion

The glioma microenvironment encompasses the surrounding tissue and cellular milieu that interacts with tumor cells during glioma growth and progression. This environment includes blood vessels, immune cells, neurons, and glial cells. Changes in the microenvironment can impact glioma development, spread, and response to treatment. Therefore, investigating the interactions of various biological processes within the microenvironment is essential for understanding glioma pathogenesis and developing novel therapeutic approaches. Current research indicates a close relationship between the glioma microenvironment and multi-omics studies, with each reinforcing the other. Changes in the microenvironment can influence gene expression and metabolic pathways in tumors, while multi-omics analyses can elucidate the molecular mechanisms underlying these alterations. Therefore, integrating microenvironmental and multi-omics investigations can yield a more holistic understanding of the pathophysiological features of gliomas, laying the groundwork for personalized treatment and precision medicine.

## Abbreviations

GBM, Glioblastoma; CyTOF, Cytometry by time-of-flight; TME, tumor microenvironment; ICB, Immune checkpoint blockade; GSCs, Glioblastoma stem cells; NPC-like, neural progenitor cells; OPC-like, oligodendrocyte progenitor cells; AC-like, astrocytes cells; MES-like, mesenchymal stromal cells; GAMs, glioma-associated macrophages and microglia; Chimeric Antigen Receptor T-Cell Immunotherapy, CAR-T; endothelial cell, EC.
